# Elevated serum uric acid to creatinine ratio is associated with adverse pregnancy outcomes: a prospective birth cohort study

**DOI:** 10.7150/ijms.95313

**Published:** 2024-06-11

**Authors:** Enjie Zhang, Shaofei Su, Shen Gao, Yue Zhang, Jiajia Wang, Jianhui Liu, Shuanghua Xie, Jinghan Yu, Qiutong Zhao, Wentao Yue, Ruixia Liu, Chenghong Yin

**Affiliations:** 1 Department of Central Laboratory, Beijing Obstetrics and Gynecology Hospital, Capital Medical University. Beijing Maternal and Child Health Care Hospital. Beijing, China.; 2 Department of Research Management, Beijing Obstetrics and Gynecology Hospital, Capital Medical University. Beijing Maternal and Child Health Care Hospital. Beijing, China.; 3 School of Public Health, Capital Medical University, Beijing, China.; 4 Laboratory for Gene-Environment and Reproductive Health, Laboratory for Clinical Medicine, Capital Medical University, Beijing, China.

**Keywords:** Serum uric acid, Serum uric acid-to-creatinine ratio, Adverse feto-maternal pregnancy outcomes

## Abstract

**Purpose:** This study evaluated the association between maternal serum uric acid-to-creatinine ratio (SUA/SCr) in the first trimester and adverse maternal and neonatal outcomes.

**Methods:** A prospective birth cohort study was conducted between 2018 and 2021. Logistic regression models and restricted cubic splines were utilized to estimate the associations between the SUA/SCr ratio and feto-maternal pregnancy outcomes. Women were stratified according to maternal age and pre-pregnancy body mass index.

**Results:** This study included 33,030 pregnant women with live singleton pregnancies. The overall prevalence of gestational diabetes mellitus (GDM), pregnancy-induced hypertension (PIH), cesarean delivery, preterm birth, large-for-gestational age (LGA), small-for-gestational age, and low Apgar scores were 15.18%, 7.96%, 37.62%, 4.93%, 9.39%, 4.79% and 0.28%, respectively. The highest quartile of SUA/SCr was associated with the highest risk of GDM (odds ratio [OR] 2.14, 95% CI 1.93-2.36), PIH (OR 1.79, 95% CI 1.58-2.04), cesarean delivery (OR 1.24, 95% CI 1.16-1.33), and preterm birth (OR 1.30, 95% CI 1.12-1.51). The associations between SUA/SCr with adverse pregnancy outcomes showed linear relationships except for GDM (P < 0.001 for all, P < 0.001 for non-linearity). Subgroup analyses revealed that the associations between the SUA/SCr ratio and the risks of PIH and LGA were significantly stronger in younger pregnant women (P = 0.033 and 0.035, respectively).

**Conclusion:** Maternal SUA/SCr levels were associated positively with the risk of adverse pregnancy outcomes. Timely monitoring of SUA and SCr levels during early pregnancy may help reduce the risk of adverse pregnancy outcomes and provide a basis for interventions.

## 1. Introduction

Serum uric acid (SUA) is the oxidation end-product of purine catabolism. Elevated SUA levels have been reported as a potential risk factor for several metabolic diseases in the general population, including diabetes, obesity, hypertension, and dyslipidemia 1-5. Increased maternal SUA levels correlated with pregnancy complications such as preeclampsia/eclampsia and gestational diabetes mellitus (GDM) 6, 7, and have adverse effects on fetal growth, and contribute to unfavorable birth outcomes, such as being preterm birth, being born small-for-gestational age (SGA), experiencing intrauterine growth restriction, having low Apgar scores, and even neonatal death 8-12. However, some inconsistencies occurred between these studies. These discrepancies may be explained by the population racial heterogeneity, the measurement of SUA levels at different time points during pregnancy, and the small sample sizes of most of these studies. Increased SUA is a consequence of renal dysfunction, but the biological and pathophysiological significance of SUA differs between patients with and without renal dysfunction 13, 14. Most previous studies did not consider the effects of renal function on the levels of SUA. Therefore, the ratio of SUA-to-serum creatinine (SUA/SCr) has recently been proposed as a more reliable indicator that is superior to SUA alone 15.

Studies have confirmed the association between the SUA/SCr ratio and metabolic syndrome and cardiovascular disease in non-pregnant population. This association is partially influenced by the modulation of blood glucose and lipid levels, body mass index (BMI), and blood pressure 15-19. For pregnant women, three studies have focused on the effect of the maternal SUA/SCr level on preeclampsia. Piani *et al.* and Yakiştiran *et al.* noted a significantly higher SUA/SCr ratio in pregnant women with preeclampsia than in controls 20, 21. Mohamed *et al.* had different results, showing a statistically nonsignificant elevation of SUA/SCr in women diagnosed with severe preeclampsia compared with those with mild preeclampsia 22. However, the association between the maternal SUA/SCr level and other adverse pregnancy outcomes was evaluated in only one of the three studies 21. Moreover, these case-control studies were conducted with small sample sizes. Thus, evidence on the relationship between maternal SUA/SCr levels and adverse maternal and neonatal outcomes is limited.

This birth cohort study was carried out with a large population as part of the China Birth Cohort Study (CBCS) 23 to analyze multiple outcomes. This study aimed to evaluate the association between maternal SUA/SCr levels during early pregnancy and adverse feto-maternal pregnancy outcomes with 33,030 pregnant women. This association was explored after stratifying participants based on maternal age and pre-pregnancy BMI.

## 2. Materials and methods

### 2.1 Study design and population

This prospective birth cohort study was conducted based on the CBCS 23. Pregnant women at 6-13^+6^ gestational weeks were enrolled in the CBCS and followed-up until pregnancy termination at Beijing Obstetrics and Gynecology Hospital, Capital Medical University, in the period between February 2018 and December 2021. These criteria were met by 40,549 pregnant women with live singletons. The study excluded participants with existing pre-pregnancy diabetes mellitus (n = 977) and hypertension (n = 1040), pregnant women with no relevant laboratory measurements (n = 3858), and those with incomplete baseline information (n = 1644). The study enrolled the remaining 33,030 pregnant women. According to previous study, odds ratios between SUA/SCr level and adverse pregnancy outcomes were 1.23-1.33 21, with the incidence rates of outcomes that significantly correlated to SUA/SCr were 4.93-37.62% in our population. The estimated sample size was 8718, that enable us to investigate the association between SUA/SCr and any of adverse pregnancy outcomes, assuming an alpha (probability of type I error) of 0.05 and delta (admissible error) of 0.10. Therefore, the actual sample size could achieve sufficient statistical power. We adhere to Declaration of Helsinki on medical protocol and ethics. Each participant signed a written informed consent. The approval of study protocol was granted by the Ethics Committee of the Beijing Obstetrics and Gynecology Hospital, Capital Medical University (#2018-KY-003-02).

### 2.2 Measurement

The baseline characteristics of participants during 6-13^+6^ gestational weeks were collected by an electronic standardized questionnaire. The information included maternal age, ethnicity, level of education, status of maternal employment, annual family income, maternal smoking and alcohol habits, maternal height and weight before pregnancy, the method of fertilization, gravidity, maternal medical history, and data of last menstrual period. Weight in kg divided by height in m^2^ was used to calculate the pre-pregnancy BMI. The pre-pregnancy BMI was categorized as normal weight (< 24 kg/m^2^), overweight (≥ 24 - < 28 kg/m^2^) or obese (≥ 28 kg/m^2^) 24. Ultrasound at baseline was used to confirm the last menstrual period.

The levels of maternal serum uric acid, creatinine, and glomerular filtration rate (eGFR) in the first trimester of pregnancy was collected from the medical records. In addition, maternal fasting blood glucose (FBG) and maternal lipid profile that included the fasting total cholesterol (TC), triglyceride (TG), high-density lipoprotein-cholesterol (HDL-C), and low-density lipoprotein-cholesterol (LDL-C) in the first trimester were also obtained from the medical records. An automated chemistry/immunology analyzer (Abbott Park, IL, USA) was used for these investigations following standard operating procedures.

Pregnancy outcomes were collected using standardized questionnaire on the day of pregnancy termination. The outcomes included GDM, pregnancy-induced hypertension (PIH), cesarean delivery, preterm birth, low Apgar score at 1, 5, and 10 minutes, large-for-gestational age (LGA), and SGA.

### 2.3 Definitions of the recorded feto-maternal pregnancy outcomes

A diagnosis of GDM was considered at a FBG > 92 mg/dL (5.1 mmol/L), glucose levels > 180 mg/dL (10.0 mmol/L) 1 h after a glucose load, or if levels were > 153 mg/dL (8.5 mmol/L) 2 h after the glucose load during the 75 g oral glucose tolerance test, as outlined by the International Association of Diabetes and Pregnancy Study Groups 25. PIH included gestational hypertension and preeclampsia-eclampsia with *de novo* hypertension occurred after 20 weeks' gestation 26. Preterm birth was characterized as a gestational age < 37 weeks at delivery 27. LGA was defined as neonatal birth weight above the sex-specific 90th percentile of gestational age 28. SGA was defined as neonatal birth weight less than the sex-specific 90th percentile of gestational age 29. A low Apgar score was determined as < 7 at 1, 5, or 10 minutes after birth 30.

### 2.4 Statistical analyses

The participants were categorized into four groups according to the SUA/SCr quartiles. Mean ± standard deviation expressed continuous variables with normal distributions, while medians (interquartile ranges) represented skewed distributions. One-way analysis of variance (ANOVA) was used to compared continuous variables with normal distributions, while the Kruskal-Wallis test was used to compare continuous variables with skewed distributions. The chi-squared test was utilized to express categorical variables.

The associations between SUA/SCr ratio and adverse pregnancy outcomes were assessed by logistic regression models. The model was adjusted for maternal age, ethnicity, the levels of maternal education, status of maternal employment, pre-pregnancy BMI, annual family income, maternal smoking and alcohol habits, the method of fertilization, first pregnancy, eGFR, FBG, TC, TG, LDL-C and HDL-C during early pregnancy. Restricted cubic splines were employed to investigate the non-linear dose-response relationships between the SUA/SCr ratio and adverse pregnancy outcomes with four knots at the 5th, 35th, 65th, and 95th percentiles. Odds ratios (OR) with 95% confidence intervals (CI) were adjusted for confounding variables. Subgroup analyses were performed after stratifying the participating women for maternal age (<35 years, ≥35 years) and pre-pregnancy BMI (≤18.5, 18.5-24, >24 kg/m^2^). A two-tailed test with *P* < 0.05 was determined statistically significant. All analyses were carried out with SAS version 9.3 (SAS Institute, Inc.; Cary, NC, USA).

## 3. Results

### 3.1 Participants' characteristics

This study included 33,030 pregnant women. Baseline characteristics according to SUA/SCr quartiles are presented (Table 1). The mean maternal age at delivery was 31.96 ± 3.83 years. With the increasing of SUA/SCr quartiles, the proportion of overweight and obese women increased significantly. Significant differences among the four quartiles of SUA/SCr were identified in maternal age, education, employment status, annual family income, pre-pregnancy BMI, fertilization method, and age at first pregnancy (all *P* < 0.001). The overall mean levels of the renal function indicators SUA, SCr and eGFR were 212.21 ± 47.78 μmol/L, 46.84 ± 6.28 μmol/L, and 139.17 ± 21.08 mL/min/1.73m^2^, respectively. The median ratio of SUA/SCr was 4.45 (3.85-5.17). All laboratory measurements, including the renal function indicators, the four blood lipid indicators (TG, TC, HDL-C and LDL-C), and FBG in the first trimester showed significant differences among the four SUA/SCr quartiles (all *P* < 0.001).

### 3.2 Outcomes

A total of 5,013 (15.18%) pregnant women developed GDM, while 2629 (7.96%) were diagnosed with PIH. The incidence of GDM and PIH increased with elevating SUA/SCr quartiles, peaking an incidence at 22.27% and 12.53% in the highest SUA/SCr quartile for GDM and PIH, respectively (Table 2). The overall incidences of cesarean delivery, preterm birth, LGA, SGA and low Apgar scores were 37.62%, 4.93%, 9.39%, 4.79% and 0.28%, respectively. Similar to GDM and PIH, the proportion of cesarean delivery, preterm birth and LGA increased significantly from quartile (Q1) to Q4 of SUA/SCr (all *P* < 0.001). The four groups did not show any significant differences in SGA and low Apgar scores (Table 2).

### 3.3 Association of SUA/SCr ratio with adverse pregnancy outcomes

**Figure 1:**
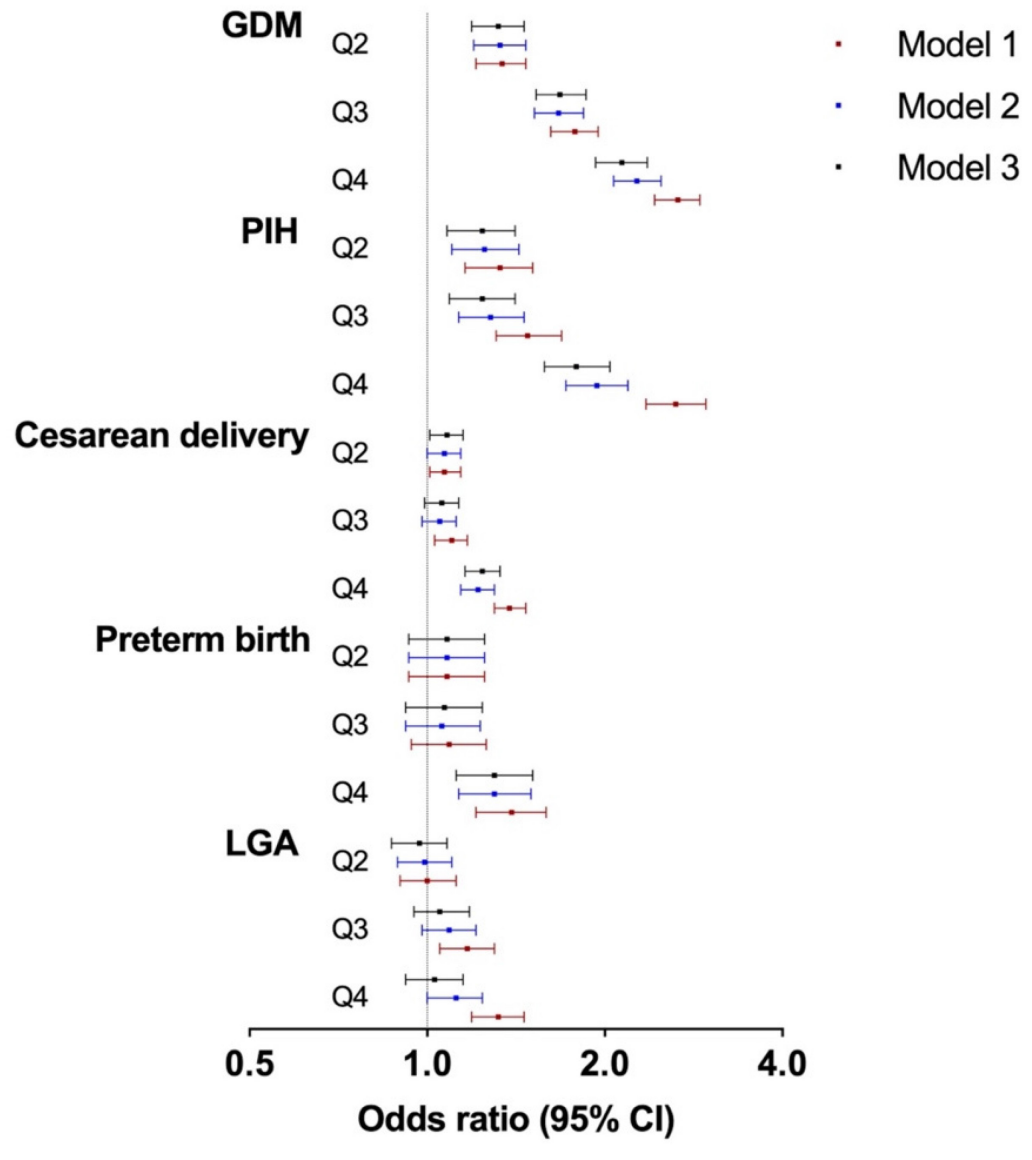
Associations between maternal SUA/SCr in the first trimester and adverse pregnancy outcomes. SUA, serum uric acid; SCr, serum creatinine; GDM, gestational diabetes mellitus; PIH, pregnancy induced hypertension; and LGA, large for gestational age. Model 1: crude; Model 2: adjusted for maternal age, ethnicity, education, employment, family annually income, current smoking, alcohol consumption, pre-pregnancy BMI, fertilization way, first pregnancy; Model 3: based on model 2, adjusted for glomerular filtration rate, fasting blood glucose, total cholesterol, triglyceride, high density lipoprotein cholesterol, low density lipoprotein cholesterol in first trimester.

The associations between maternal SUA/SCr and the risks of adverse maternal and neonatal outcomes were investigated by logistic regression models (Table 3 and Figure 1). After adjusting for confounding variables, the risk of GDM increased with increasing SUA/SCr quartile, compared with that with SUA/SCr Q1 (Q2: OR 1.32, 95% CI 1.19-1.46; Q3: OR 1.68, 95% CI 1.53-1.86; Q4: OR 2.14, 95% CI 1.93-2.36). Similarly, pregnant women with increased SUA/SCr level had a significantly higher risk of PIH (Q2: OR 1.24, 95% CI 1.08-1.41; Q3: OR 1.24, 95% CI 1.09-1.41; Q4: OR 1.79, 95% CI 1.58-2.04) than those with lower SUA/SCr ratio. Moreover, individuals within SUA/SCr Q2 and Q4 had 8% (Q2: OR 1.08, 95% CI 1.01-1.15) and 24% (OR 1.24, 95% CI 1.16-1.33) higher risk of cesarean delivery compared to those within SUA/SCr Q1, while only the highest quartile of SUA/SCr was linked with higher risk of preterm birth (Q4: OR 1.30, 95% CI 1.12-1.51). Logistic regression analysis showed no significant differences in the risk of LGA across the four SUA/SCr groups.

**Figure 2:**
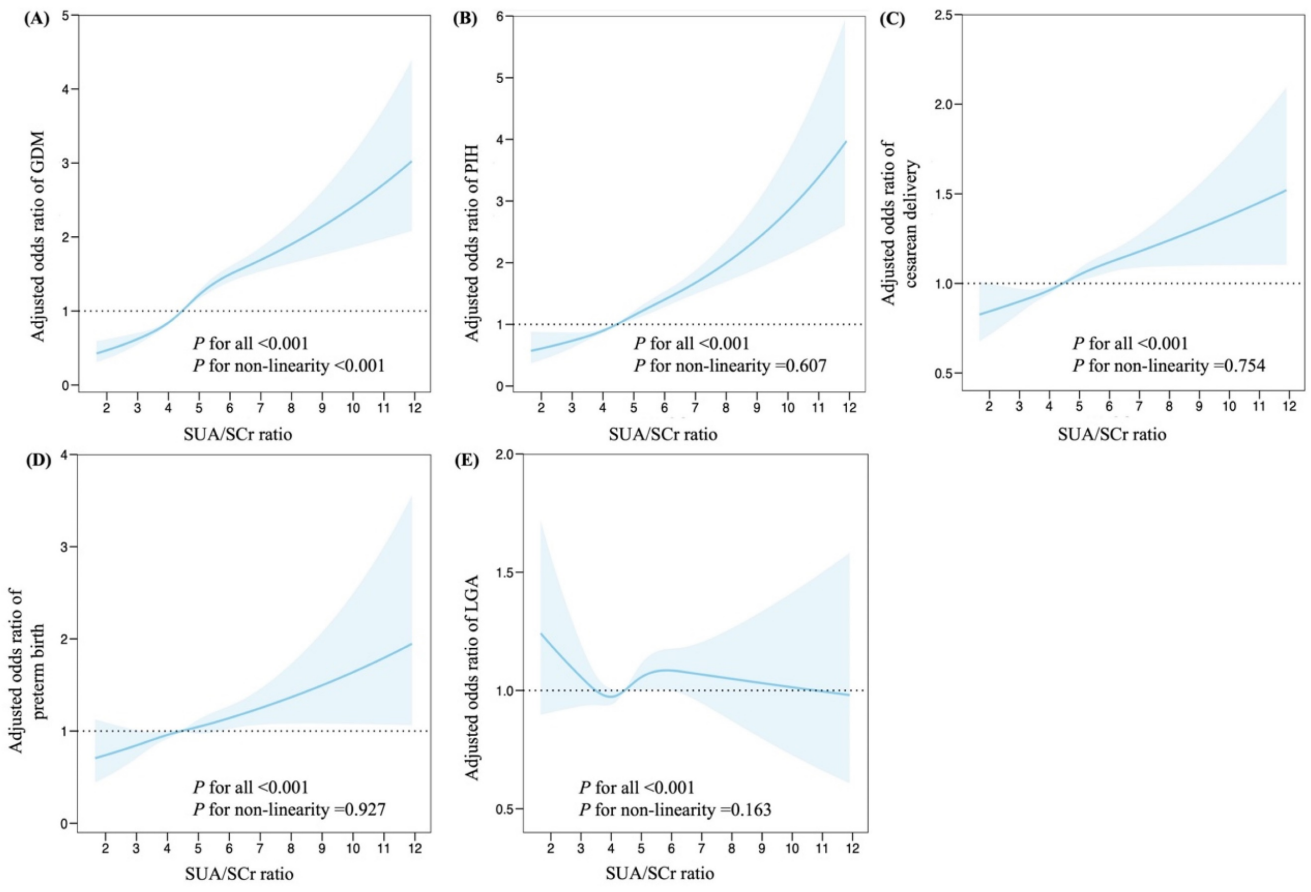
Associations between maternal SUA/SCr in the first trimester and adverse pregnancy outcomes, plotted with restricted cubic splines. Odds ratios and 95% CI were estimated using logistic regression model adjusted for the maternal age, ethnicity, education, employment, family annually income, smoking, alcohol consumption, pre-pregnancy BMI, fertilization way, first pregnancy, glomerular filtration rate, fasting blood glucose, total cholesterol, triglyceride, high density lipoprotein cholesterol, low density lipoprotein cholesterol in the first trimester. SUA, serum uric acid; SCr, serum creatinine; GDM, gestational diabetes mellitus; PIH, pregnancy induced hypertension; and LGA, large for gestational age.

Further investigation with restricted cubic splines were conducted to evaluate dose-response relationships between maternal SUA/SCr and adverse pregnancy outcomes (Figures 2). The spline model, adjusted for multiple variables, demonstrated a non-linear relationship between maternal SUA/SCr ratio and the risk of GDM (*P* < 0.001 for all, *P* < 0.001 for non-linearity, Figure 2A). Maternal SUA/SCr were linearly associated with the risk of PIH (*P* < 0.001 for all, *P* = 0.607 for non-linearity), cesarean delivery (*P* < 0.001 for all, *P* = 0.754 for non-linearity), preterm birth (*P* < 0.001 for all, *P* for = 0.927 non-linearity), and LGA (*P* < 0.001 for all, *P* = 0.163 for non-linearity, Figure B-E).

### 3.4 Subgroup analysis between SUA/SCr and adverse pregnancy outcomes

Subgroup analyses were carried out after stratification according to maternal age and pre-pregnancy BMI (Table 4). The results demonstrated that the relationships between the SUA/SCr ratio in the first trimester and the risks of PIH and LGA were significantly stronger in younger pregnant women (*P* = 0.033 and 0.035 respectively). The correlations between SUA/SCr ratio and maternal age were confirmed, showing no effects on the incidence risk of GDM development, cesarean delivery, and preterm birth (all *P* > 0.05). The associations between maternal SUA/SCr levels and risk of adverse outcomes were consistent among the different pre-pregnancy BMI subgroups (all *P* > 0.05).

## 4. Discussion

This prospective birth cohort study identified that higher maternal SUA/SCr levels during early pregnancy were associated with a higher risk of adverse pregnancy outcomes, including GDM, PIH, cesarean delivery, LGA, and preterm birth, compared with the lowest SUA/SCr quartile. These associations showed linear relationships, except for GDM, which demonstrated a non-linear dose-response relationship. These associations were consistent across the different pre-pregnancy BMI subgroups, and the associations between maternal SUA/SCr levels and the risks of PIH and LGA were stronger in younger pregnant women.

Maternal SUA levels vary at different stages of a normal pregnancy. The concentration of SUA decreased significantly during early pregnancy in healthy pregnant women, stabilizing until the 24^th^ gestational week 31, 32. The mean value of SUA (212.21 ± 47.78 µmol/L) in the first trimester for all participants in present study was similar to that of a cohort study of 85,609 pregnant women (198.0 [172.0 - 227.0] µmol/L) during 7-12 weeks of gestation 33. The absolute value of SUA/SCr was 4.45 for the overall pregnant population in the first trimester in our study. Only one previous study reported a value for the first trimester of pregnancy (approximately 4 for controls, and 7 for preeclampsia) 21.

SUA/SCr is considered a functionally normalized renal SUA and has been proposed as a superior biomarker 15. The general population is at a higher risk of metabolic syndrome with an increased SUA/SCr ratio 15-17. The incidences of hypertension, diabetes, and cardiovascular disease increase substantially with increasing SUA/SCr quartiles 15. This is consistent with our findings showing that the proportion of GDM, PIH, cesarean delivery, preterm birth and LGA were significantly increased from Q1 to Q4 of SUA/SCr during the early pregnancy. Three case-control studies of relatively small sample sizes have investigated the link between SUA/SCr ratio and pregnancy complications, mainly preeclampsia 20-22. Piani *et al.* analyzed the correlation between maternal SUA/SCr levels and adverse outcomes in all trimesters of pregnancy. They showed that only SUA/SCr in third trimester correlated positively with the risk of developing preeclampsia, being preterm birth, and experiencing composite neonatal outcomes, such as low weight at birth, need for neonatal intensive care, and mortality 21. Another study with 170 participants showed a low-to-moderate correlation between SUA/SCr and the neonatal platelet-to-lymphocyte ratio 20. Our study estimated the association of maternal SUA/SCr levels in the first trimester with maternal and neonatal outcomes, including GDM, PIH, cesarean delivery, preterm birth, LGA, SGA and low Apgar score. After adjustment, a significant association was found in GDM, PIH, cesarean delivery and preterm birth. However, a cross-sectional study including 23 patients with preeclampsia into mild preeclampsia and 22 with severe preeclampsia found no significant relationship between SUA/SCr and severity of preeclampsia 22. The inconsistency between these studies may be attributed to the variations in their study designs, populations, and sample sizes. Further prospective studies throughout all trimester of pregnancy are required to confirm and validate our findings.

The spline analysis in our study showed a non-linear dose-response relationship between maternal SUA/SCr during the early pregnancy and the risk of GDM. This finding is consistent with a cohort study involving 85,609 pregnant women, that showed a non-linear correlation between the levels of SUA and GDM. Further analysis by interval of gestational weeks maintained the same results for early pregnancy at 7-12 weeks gestation 33. In contrast, a meta-analysis of 23 studies and 105,380 participants showed a linear dose-response relationship between SUA levels and GMD 34. For hypertensive disorders of pregnancy, the SUA level is a reliable predictor of preeclampsia 35. A retrospective cohort study of 4725 Chinese pregnant women showed that maternal SUA levels before 20 weeks of gestation increased the risk of preeclampsia, especially during the first 8-12 weeks of gestation 36. Another retrospective study of 780 pregnant women with advanced maternal age revealed a significant association between SUA levels measured in early pregnancy and pregnancy-induced hypertensive (gestational hypertension and preeclampsia) 37. Similarly, our study found that elevated SUA/SCr ratio during the first trimester significantly increased the risk of developing PIH, consistent with another study conducted in the third trimester of pregnancy 21. For preterm birth, a meta-analysis including three studies with 5358 participants reported a significant correlation between maternal SUA levels during pregnancy and preterm birth, with an OR of 2.52 12. A previous study found that increasing SUA/SCr in the third trimester increased the likelihood of preterm birth by 23% 21. Our research is consistent with this observation, pregnant women within the SUA/SCr Q4 in the first trimester had a 30% higher risk of preterm birth. Regarding SGA, a retrospective cohort study in China with 23,194 participants observed a “J-shaped” relationship between maternal SUA in the third trimester and SGA. They also found maternal SUA levels in both first and third trimesters significantly affected the development of SGA 38. In our research, we did not observe any notable differences in the association between the SUA/SCr quartiles in the first trimester and SGA. In a prospective analysis of 1,541 pregnant women, Laughon *et al.* showed that increased SUA levels during the early pregnancy were not associated with SGA 39. Elevated SUA/SCr ratio during early pregnancy increase the risk of adverse pregnancy and neonatal outcomes. Prenatal screening and prevention before and during early stages of pregnancy are particularly important, especially in women with chronic diseases requiring medication adjustment 40.

According to our subgroup analysis, younger pregnant women with elevated SUA/SCr levels had a greater incidence risk of PIH and LGA. For GDM, our subgroup analysis suggested that the association between SUA/SCr ratio in the first trimester and the incidence risk of GDM was similar among the different pre-pregnancy BMI and maternal age subgroups. Similar results were obtained in a study, reporting that the association between increased maternal SUA levels and the risk of GDM was independent of the pre-pregnancy BMI 33. However, Duo *et al.* suggested this association was stronger in women with pre-pregnancy BMI ≥ 24kg/m^2^ 41. In age-stratified analyses, Zhao *et al.* and Duo *et al.* showed that pregnant women ≥ 35 years were more likely to develop GDM 33, 41. Other studies have reported that this association was stronger in younger pregnant women with elevated SUA levels 34, 42.

The mechanisms by which elevated SUA/SCr levels increase the risk of adverse pregnancy outcomes are not fully understood. SUA/SCr levels are associated with metabolic factors 16-18. Elevated SUA levels inhibit insulin signal, cause insulin resistance, and decrease glucose uptake 43. Moreover, high SUA/SCr ratios are associated with inflammation and oxidative stress, which cause senescence, apoptosis, and mitochondrial dysfunction in endothelial cells. Expression of the NLRP3 inflammasome is increased in the placentas in late-onset preeclampsia 44, and leading to activation of nuclear factor kappa-B and extracellular signal-regulated kinase signaling pathways 45, 46. These mechanisms can cause diabetes and hypertension. In addition, increasing maternal SUA levels could attenuate trophoblast invasion and disturb spiral artery formation, contributing to placental hypoxia and abnormal placenta development 47. Further studies are required to clarify biological mechanisms responsible for the SUA/SCr ratio in the increased risk of adverse pregnancy outcomes.

This prospective cohort study is the primary one with a substantial sample size to comprehensively assess the association between maternal SUA/SCr ratio in early pregnancy and the risk of adverse pregnancy outcomes. However, several limitations exist in present study. Although this study conducted with a large sample size, it was conducted in a single-center; therefore, it was not nationally representative. Second, maternal SUA and SCr were collected only in first trimester of pregnancy, since they were not available in the other trimesters. Third, this study included only the pregnancy-induced hypertension occurred after 20 weeks' gestation, encompassing gestational hypertension and preeclampsia-eclampsia. Fourth, only pregnant women with live singletons were included in this study; further studies could include all pregnancy losses. Fifth, although we adjusted for many potential confounders, the analysis did not include several potential variables, including maternal physical activity and diet. Finally, the participants self-reported on a questionnaire; however, the questions were presented in simple language and guided by trained researchers, doctors or nurses.

## 5. Conclusion

Our analyses of this large cohort showed that an elevated SUA/SCr ratio in the first trimester of pregnancy increased the risk of adverse maternal and neonatal outcomes. These feto-maternal outcomes correspond to the nature of the association before and after stratification for women's age and pre-pregnancy BMI. Timely monitoring SUA and SCr levels during early pregnancy may help reduce the risk of development of adverse pregnancy outcomes, and provide a basis for interventions.

## Figures and Tables

**Table 1:** Characteristics of participants according to quartiles of SUA/SCr ratio in the present study.

Variables	Total (N=33,030)	Quartiles of SUA/SCr in first trimester of pregnancy				
		Q1 (N=8,256)	Q2 (N=8,259)	Q3 (N=8,257)	Q4 (N=8,258)	P value
Maternal age at delivery, years	31.96±3.83	32.25±3.81	31.86±3.77	31.91±3.85	31.81±3.87	<0.001
Maternal Han ethnicity	30,531 (92.43)	7,640 (92.54)	7,638 (92.48)	7,630 (92.41)	7,623 (92.31)	0.951
Maternal education						<0.001
< College	2,003 (6.06)	417 (5.05)	418 (5.06)	497 (6.02)	671 (8.13)	
Undergraduate/College	22,759 (68.90)	5,467 (66.22)	5,666 (68.60)	5,760 (69.76)	5,866 (71.03)	
> Postgraduate or higher	8,268 (25.03)	2,372 (28.73)	2,175 (26.33)	2,000 (24.22)	1,721 (20.84)	
Maternal unemployment	2,340 (7.08)	523 (6.33)	553 (6.70)	573 (6.94)	691 (8.37)	<0.001
Family annually income, Yuan						<0.001
<100000	3,278 (9.92)	684 (8.28)	745 (9.02)	879 (10.65)	970 (11.75)	
100000-400000	20,771 (62.89)	5,204 (63.03)	5,221 (63.22)	5,162 (62.52)	5,184 (62.78)	
>400000	8,981 (27.19)	2,368 (28.68)	2,293 (27.76)	2,216 (26.84)	2,104 (25.48)	
Maternal current smoking	63 (0.19)	15 (0.18)	12 (0.15)	12 (0.15)	24 (0.29)	0.104
Maternal alcohol consumption	1,410 (4.27)	352 (4.26)	371 (4.49)	339 (4.11)	348 (4.21)	0.658
Pre-pregnancy BMI (kg/m^2^)						<0.001
<24	26,638 (80.65)	7,413 (89.79)	7,074 (85.65)	6,576 (79.64)	5,575 (67.51)	
24-27.9	5,065 (15.33)	739 (8.95)	1,029 (12.46)	1,369 (16.58)	1,928 (23.35)	
≥28	1,327 (4.02)	104 (1.26)	156 (1.89)	312 (3.78)	755 (9.14)	
Natural pregnancy	30,949 (93.70)	7,782 (94.26)	7,791 (94.33)	7,753 (93.90)	7,623 (92.31)	<0.001
First pregnancy	17,693 (53.57)	4,227 (51.20)	4,496 (54.44)	4,464 (54.06)	4,506 (54.57)	<0.001
SUA, μmol/L	212.21±47.78	166.62±26.69	197.85±24.50	221.05±29.07	263.31±44.72	<0.001
SCr, μmol/L	46.84±6.28	49.44±6.15	47.67±5.73	46.26±5.88	43.99±6.04	<0.001
SUA/SCr	4.45 (3.85-5.17)	3.46 (3.18-3.67)	4.15 (4.00-4.30)	4.77 (4.60-4.95)	5.76 (5.43-6.33)	<0.001
eGFR, ml/min/1.73m^2^	139.17±21.08	133.25±18.35	137.46±19.33	140.57±20.84	145.39±23.55	<0.001
FBG, mmol/L	4.64±0.35	4.65±0.34	4.64±0.34	4.62±0.35	4.65±0.38	<0.001
TG, mmol/L	0.97 (0.76-1.26)	0.88 (0.71-1.11)	0.94 (0.74-1.19)	0.99 (0.78-1.28)	1.10 (0.85-1.47)	<0.001
TC, mmol/L	4.21±0.68	4.11±0.65	4.18±0.67	4.22±0.68	4.33±0.72	<0.001
HDL-C, mmol/L	1.51±0.29	1.55±0.28	1.53±0.29	1.51±0.29	1.47±0.30	<0.001
LDL-C, mmol/L	2.22±0.59	2.12±0.56	2.18±0.56	2.23±0.59	2.35±0.63	<0.001

Note: SUA, serum uric acid; SCr, serum creatinine; BMI, body mass index; eGFR, glomerular filtration rate; FBG, fasting blood glucose; TG, triglyceride; TC, total cholesterol; HDL-C, high density lipoprotein cholesterol; and LDL-C, low density lipoprotein cholesterol.

**Table 2:** Pregnancy and neonatal outcomes according to quartiles of SUA/SCr ratio in the present study.

Outcomes	Total (N=33,030)	Quartiles of SUA/SCr in first trimester of pregnancy				
		Q1 (N=8,256)	Q2 (N=8,259)	Q3 (N=8,257)	Q4 (N=8,258)	P value
GDM	5,013 (15.18)	804 (9.74)	1,040 (12.59)	1,330 (16.11)	1,839 (22.27)	<0.001
PIH	2,629 (7.96)	425 (5.15)	554 (6.71)	615 (7.45)	1,035 (12.53)	<0.001
Cesarean delivery	12,425 (37.62)	2,870 (34.76)	3,005 (36.38)	3,049 (36.93)	3,501 (42.40)	<0.001
Preterm birth	1,628 (4.93)	360 (4.36)	386 (4.67)	391 (4.74)	491 (5.95)	<0.001
LGA	3,101 (9.39)	699 (8.47)	701 (8.49)	804 (9.74)	897 (10.86)	<0.001
SGA	1,582 (4.79)	385 (4.66)	418 (5.06)	396 (4.80)	383 (4.64)	0.564
Low Apgar score	94 (0.28)	26 (0.31)	16 (0.19)	24 (0.29)	28 (0.34)	0.315

Note: GDM, gestational diabetes mellitus; PIH, pregnancy induced hypertension; LGA, large-for-gestational age; and SGA, small-for-gestational age.

**Table 3:** Associations between quartiles of SUA/ SCr in first trimester and adverse pregnancy outcomes.

Outcomes	Odds ratio (95% CI)			
	Quartile 1	Quartile 2	Quartile 3	Quartile 4
GDM				
Model 1	Reference	1.34 (1.21-1.47)	1.78 (1.62-1.95)	2.66 (2.43-2.90)
Model 2	Reference	1.33 (1.20-1.47)	1.67 (1.52-1.84)	2.27 (2.07-2.49)
Model 3	Reference	1.32 (1.19-1.46)	1.68 (1.53-1.86)	2.14 (1.93-2.36)
PIH				
Model 1	Reference	1.33 (1.16-1.51)	1.48 (1.31-1.69)	2.64 (2.35-2.97)
Model 2	Reference	1.25 (1.10-1.43)	1.28 (1.13-1.46)	1.94 (1.72-2.19)
Model 3	Reference	1.24 (1.08-1.41)	1.24 (1.09-1.41)	1.79 (1.58-2.04)
Cesarean delivery				
Model 1	Reference	1.07 (1.01-1.14)	1.10 (1.03-1.17)	1.38 (1.30-1.47)
Model 2	Reference	1.07 (1.00-1.14)	1.05 (0.98-1.12)	1.22 (1.14-1.30)
Model 3	Reference	1.08 (1.01-1.15)	1.06 (0.99-1.13)	1.24 (1.16-1.33)
Preterm birth				
Model 1	Reference	1.08 (0.93-1.25)	1.09 (0.94-1.26)	1.39 (1.21-1.59)
Model 2	Reference	1.08 (0.93-1.25)	1.06 (0.92-1.23)	1.30 (1.13-1.50)
Model 3	Reference	1.08 (0.93-1.25)	1.07 (0.92-1.24)	1.30 (1.12-1.51)
LGA				
Model 1	Reference	1.00 (0.90-1.12)	1.17 (1.05-1.30)	1.32 (1.19-1.46)
Model 2	Reference	0.99 (0.89-1.10)	1.09 (0.98-1.21)	1.12 (1.00-1.24)
Model 3	Reference	0.97 (0.87-1.08)	1.05 (0.95-1.18)	1.03 (0.92-1.15)

Note: SUA, serum uric acid; SCr, serum creatinine; GDM, gestational diabetes mellitus; PIH, pregnancy induced hypertension; and LGA, large-for-gestational age. Model 1: crude; Model 2: adjusted for maternal age, ethnicity, education, employment, family annually income, current smoking, alcohol consumption, pre-pregnancy BMI, fertilization way, first pregnancy; Model 3: based on model 2, adjusted for glomerular filtration rate, fasting blood glucose, total cholesterol, triglyceride, high density lipoprotein cholesterol, low density lipoprotein cholesterol in first trimester.

**Table 4:** Adjusted ORs and 95% CI for the association between high SUA/ SCr (Q4) in first trimester and risk of adverse pregnancy outcomes, stratified by maternal age and pre-pregnancy BMI.

Variables	Maternal age at delivery		*P* for interaction	Pre-pregnancy BMI (kg/m^2^)			*P* for interaction
	< 35 years	≥ 35 years		< 24	24-27.9	≥ 28	
GDM	2.09 (1.85-2.37)	2.19 (1.84-2.59)	0.833	2.09 (1.86-2.34)	2.09 (1.66-2.64)	1.71 (1.02-2.88)	0.215
PIH	1.94 (1.66-2.26)	1.52 (1.21-1.91)	0.033	1.68 (1.44-1.96)	1.793 (1.36-2.37)	3.29 (1.74-6.22)	0.153
Cesarean delivery	1.30 (1.20-1.41)	1.08 (0.94-1.23)	0.065	1.22 (1.13-1.32)	1.20 (1.01-1.44)	1.49 (0.96-2.30)	0.591
Preterm birth	1.24 (1.03-1.49)	1.45 (1.12-1.89)	0.387	1.26 (1.06-1.49)	1.53 (1.04-2.25)	0.89 (0.41-1.94)	0.373
LGA	1.24 (1.03-1.49)	0.89 (0.72-1.10)	0.035	1.04 (0.91-1.189)	1.04 (0.81-1.35)	0.98 (0.55-1.76)	0.950

Note: SUA, serum uric acid; SCr, serum creatinine; GDM, gestational diabetes mellitus; PIH, pregnancy induced hypertension; LGA, large-for-gestational age. Adjusted for maternal age, ethnicity, education, employment, family annually income, smoking, alcohol consumption, pre-pregnancy BMI, fertilization way, first pregnancy, glomerular filtration rate, fasting blood glucose, total cholesterol, triglyceride, high density lipoprotein cholesterol, low density lipoprotein cholesterol in first trimester.

## Abbreviations

ANOVA: analysis of variance
BMI: body mass index
CBCS: China Birth Cohort Study
eGFR: glomerular filtration rate
FBG: fasting blood glucose
GDM: gestational diabetes mellitus
HDL-C: high-density lipoprotein cholesterol
LDL-C: low-density lipoprotein cholesterol
LGA: large-for-gestational age
OR: odds ratio
PIH: pregnancy-induced hypertension
SGA: small-for-gestational age
SUA: serum uric acid
SUA/SCr: serum uric acid-to-creatinine ratio
TG: triglyceride
TC: total cholesterol
